# Fatal cold agglutinin-induced haemolytic anaemia: a case report

**DOI:** 10.1186/1752-1947-4-252

**Published:** 2010-08-06

**Authors:** Gianluca Lodi, Daniela Resca, Roberto Reverberi

**Affiliations:** 1 Blood Transfusion Service - Arcispedale S. Anna, 203 C.so Giovecca, 44100 Ferrara - Italy

## Abstract

**Introduction:**

Cold agglutinin disease usually develops as a result of the production of a specific immunoglobulin M auto-antibody directed against the I/i and H antigens, precursors of the ABH and Lewis blood group substances, on red blood cells. Autoimmune and lymphoproliferative disorders, *Mycoplasma pneumoniae *and other infections can be associated with the production of cold agglutinins. In its classic presentation with haemolytic anaemia and Raynaud's syndrome, cold agglutinin disease is usually idiopathic. Several factors play a role in determining the ability of a cold agglutinin to induce a haemolytic anaemia such as antibody concentration and temperature range, in particular the highest temperature at which antibodies interact with red blood cells.

**Case presentation:**

A 48-year-old Caucasian man presented to our hospital with symptoms of extreme asthenia caused by severe anaemia. The transfusion of red blood cells (O Rh-positive), started as prescribed by the emergency guidelines in force without pre-transfusion tests, induced fatal haemolysis because of the presence of high levels of anti-H antibodies in his blood, that reacted with the large amount of H antigen in universal (0) red blood cells.

**Conclusion:**

Emergency transfusion of universal red blood cells (0 Rh-positive or negative) is usually accepted by the international guidelines in force in emergency departments. In this report we describe a rare complication caused by the very high concentration in the recipient of cold agglutinins and the activation of the complement system, responsible for red blood cell lysis and consequent fatal cardiovascular shock. We conclude that emergency transfusion of universal red blood cells (0 Rh-positive or negative) may be dangerous and its risk should be assessed against the risk of delaying transfusion until the pre-transfusion tests are completed.

## Introduction

Cold agglutinins were first described by Landsteiner in 1903 [1]. Their pathological action against red blood cells (haemolytic anaemia) and blood vessels (Raynaud's syndrome) was described some years later by Clough and Iwai [2,3]. In 1953 Schubothe coined the term: Cold Agglutinin Disease (CAD) [4].

CAD is characterized by an auto-antibody [5] which is able to agglutinate red blood cells (RBCs) at temperatures lower than that of the body, and subsequently to activate the complement system responsible for lysis of RBCs.

Patients show haemolytic anaemia of varying degrees of severity, as well as episodes of hemoglobinuria and acrocyanosis, which arise or worsen upon exposure to low temperatures.

Cold agglutinin antibodies are mainly specific for the I/i and H RBCs membrane systems [6], and their production can be stimulated by *Mycoplasma pneumoniae *or infection by the Epstein-Barr virus, as well as by lymphoproliferative disorders such as Waldenström's macroglobulinemia.

The auto-antibody involved is usually an IgM, less frequently an IgA or IgG, which is able to agglutinate RBCs at temperatures of between 0 and 5°C. Complement activation generally occurs between 20 and 25°C, but is also possible at normal body temperature. It is also important to note that agglutination is not necessary for complement activation, especially in patients with high levels of auto-antibodies (wide thermal range of cold agglutinins) [7,8]. This obviously has serious repercussions in a clinical setting.

## Case presentation

A 48-year-old Caucasian man presented to the Accident and Emergency Department of our hospital with symptoms of extreme asthenia, but showed no evidence of Raynaud's syndrome. In the past few months, he had complained about a productive cough and post-prandial vomiting. At admission, he was evidently dehydrated and undernourished, very pale, dyspnoeic and tachycardiac (110 bpm) at rest. Heart sounds were soft but no other pathologic sign concerning his lungs and abdomen was noted. His blood pressure was 80 over 50 mmHg.

A blood cell count showed severe anaemia (haemoglobin = 3.8gr/dl) and the patient was prescribed an emergency transfusion of RBCs (0 Rh-positive), owing to the severe anaemia associated with dyspnoea and tachycardia at rest, and hypotension. Blood samples were also sent to our Blood Transfusion Service at this time. Previous data relating to our patient was not found in our records.

After centrifugation, samples showed low hematocrit and normal plasma appearance. The direct blood group test resulted in unequivocally A with Rh phenotype Ccddee, while the indirect test revealed agglutination of B cells and a strong agglutination of 0 cells. Antibody screening also showed strong agglutination (4+) of all panel cells.

The above-mentioned Accident and Emergency Department was immediately alerted to our patient's immunohaematological situation, and we advised urgent cessation of the transfusion of RBCs (0 Rh-positive), which the physician had already initiated as prescribed by the emergency guidelines in force. We also recommended our patient's transferral to the Haematology Department in Ferrara City Hospital, where he arrived in a state of severe cardiovascular shock.

Blood samples taken from him at this time showed dramatic haemolysis, which led to his death within a few hours.

Subsequent blood tests revealed the presence of cold agglutinin syndrome with very high levels of anti-H (1:65.600). He showed positive (3+) results for the direct antiglobulin test for complement fractions, which caused the intravascular haemolysis and consequent cardiovascular shock.

His death prevented further attempts to define the aetiology of his condition. However, an emergency ultrasonography had shown several enlarged lymph nodes (average diameter, 5 cm) along the iliac vessels and the thoracic and abdominal aorta, suggesting a lymphomatous pathology.

## Conclusion

The dramatic clinical situation of our patient upon presentation led the physician to commence emergency transfusion of universal RBCs (0 Rh-positive) according to the guidelines which were designed to safeguard against major complications such as multi-organ failure due to severe anaemia.

However, the transfusion of 0 RBCs, expressing a large amount of H antigens, caused dramatic haemolysis, cardiovascular shock and our patient's death within a few hours.

We conclude that emergency transfusion of universal red blood cells (0 Rh-positive/negative) may be dangerous and the risks of the procedure should be assessed against the risks of delaying transfusion until the pre-transfusion tests are completed.

## Competing interests

The authors declare that they have no competing interests.

## Authors' contributions

GL and DR performed pre-transfusion evaluations and made the diagnosis; then they searched the literature for other similar reports. GL and RR drafted the manuscript. All authors read and approved the final manuscript.

## Consent

Written consent was obtained from our patient's next-of-kin for publication of this case study. A copy of the written consent is available for review by the Editor-in-Chief of this journal.
